# Metformin as a tool to control antipsychotic-induced metabolic syndrome - case report

**DOI:** 10.1192/j.eurpsy.2023.2144

**Published:** 2023-07-19

**Authors:** L. Horosan, D. E. Nistor, I. Paunica, R. A. Stoica

**Affiliations:** 1 Psychiatry, Prof. Dr. Alexandru Obregia Psychiatry Hospital; 2 Diabetes Nutrition and Metabolic Disease, Prof. Dr. N. Paulescu Institute of Diabetes Nutrition and Metabolic Disease, Bucharest, Romania

## Abstract

**Introduction:**

The decreased capacity of testing reality causes patients with psychosis consequences regarding their families, professional life, and social interactions, with an overall reduction in quality of life. In these cases, antipsychotic treatment is mandatory to recreate the patient’s connection with the environment. Second-generation antipsychotics (SGAs), particularly clozapine and olanzapine, can have severe metabolic side effects that impact body weight, insulin resistance, and glucose metabolism. The specific mechanism that determines such metabolic processes is not yet fully understood. Recent research has demonstrated that metformin may be utilized to regulate metabolic processes. The ultimate purpose of using this adjunctive therapy is to effectively control both physical and mental health difficulties among psychiatric patients.

**Objectives:**

The primary purpose of this report is to underline the importance of adverse metabolic reactions of antipsychotics and to study the effectiveness of metformin regarding this matter.

**Methods:**

Our patient is a 33 years-old man who was diagnosed with schizoaffective disorder around the age of 32. He was initially treated with olanzapine; during the first year, he gained more than 20kg. Severe weight gain was a significant health factor that determined us to search for therapeutic alternatives. Metformin was added, monitoring BMI and abdominal circumference. Because of the severe body weight gain, switching from olanzapine to aripiprazole was attempted, but the psychiatric symptoms worsened. Paliperidone was considered and administered, concomitant with rising doses of metformin. Although an initial increase in body weight was documented when paliperidone was administered, his body weight deescalated significantly after metformin reached a therapeutic dose of 2000mg per day.

**Results:**

Metformin co-administered with antipsychotic medication helped to control the severe metabolic adverse effects in this case. Reaching a lower BMI index after adding metformin to paliperidone was a therapeutic goal and essential for the patient’s physical and psychological health.

**Conclusions:**

Metformin is a complex treatment widely prescribed as an antidiabetic drug. Lately, attention has shifted towards its effects on controlling the adverse metabolic effects of antipsychotics. This case underlines the importance of the metabolic syndrome as an adverse reaction of the SGAs and presents the results of this treatment option for schizoaffective disorder treated with antipsychotics. Although the current recommendation is to switch to another antipsychotic with lower metabolic risk, the new drug may not control the psychiatric symptoms in all cases. Therefore, metformin is an adjuvant solution in situations where antipsychotic treatment can cause severe metabolic reactions with a significant impact on the patient’s physical health.

**Disclosure of Interest:**

None Declared

